# THE RELATIONSHIP BETWEEN 10-YEAR CHANGES IN COGNITIVE ABILITY AND SUBSEQUENT MORTALITY: FINDINGS FROM THE ACTIVE

**DOI:** 10.1093/geroni/igad104.1297

**Published:** 2023-12-21

**Authors:** Diefei Chen, Alden Gross, Sherry Willis, George Rebok

**Affiliations:** Johns Hopkins, Baltimore, Maryland, United States; Johns Hopkins University Bloomberg School of Public Health, Baltimore, Maryland, United States; University of Washington, Seattle, Washington, United States; Johns Hopkins University, Baltimore, Maryland, United States

## Abstract

Cognitive ability and cognitive decline have been linked with mortality in older adults. However, little was understood about the role of cognitive interventions on mortality outcomes in this population. Using twenty-year follow-up data from the Advanced Cognitive Training for Independent and Vital Elderly (ACTIVE) trial, we examined the association between cognitive change and mortality risk, and the effect of ACTIVE cognitive training (memory, reasoning, and speed of processing) on mortality risk. Mortality was ascertained through linkage to the National Death Index database. To model time to death as a function of cognitive change and training effect, we used shared growth-survival models with simultaneously estimated latent intercepts and slopes as predictors. Among the 2802 participants, 2021 died on or before the year 2019 (72.1%). Both higher baseline level and slower decline in global cognition were associated with lower hazards of all-cause mortality after adjusting for covariates (HR = 0.68, 95% CI 0.58, 0.79; HR = 0.42, 95% CI 0.40, 0.44, respectively). We did not observe any significant effects of ACTIVE cognitive training in memory, reasoning, or speed of processing on all-cause mortality. Our findings demonstrated the association between the trajectory of cognitive change and mortality among older adults, independent of cognitive training interventions. More work is needed to identify relevant timing as well as modalities of non-pharmaceutical interventions that can promote healthy longevity.

